# Responses in shoot elongation, carbohydrate utilization and growth recovery of an invasive species to submergence at different water temperatures

**DOI:** 10.1038/s41598-017-18735-7

**Published:** 2018-01-10

**Authors:** Xiao qi Ye, Bo Zeng, Jin liu Meng, Ming Wu, Xiao ping Zhang

**Affiliations:** 1 Institute of Subtropical Forestry, Chinese Academy of Forestry/Research Station of Hangzhou Bay Wetlands Ecosystem, National Forestry Bureau, Fuyang, China; 2 Key Laboratory of Eco-environments in Three Gorges Reservoir Region (Ministry of Education), Chongqing Key Laboratory of Plant Ecology and Resources in Three Gorges Reservoir Region, School of Life Sciences, Southwest University, Chongqing, China

## Abstract

Widely distributed amphibious exotic plant species may respond plastically to water temperatures when submerged. *Alternanthera philoxeroides*, a highly flood-tolerant species, originates from tropical regions and has successfully invaded temperate regions. The wide distribution of this species suggests it can respond to flooding at different water temperatures. In this study, the plastic responses of *A. philoxeroides* plants to submergence at water temperatures of 10 °C, 20 °C and 30 °C were investigated. The *A. philoxeroides* plants had large pools of non-structural carbohydrates, which were readily mobilized upon submergence. Submergence hindered biomass accumulation and decreased the carbohydrate content level and respiration rate (P < 0.05). Water temperature had remarkable effects on shoot elongation, carbohydrate utilization and recovery growth. With decreasing water temperature, the respiration rate was lower and carbohydrate content decreased more slowly, but the post-submergence biomass accumulation was faster (P < 0.05), indicating a beneficial effect of low water temperature for recovery. However, high water temperatures accelerated shoot elongation (P < 0.05), which benefitted the submerged plants more if contact with air was restored. These results suggest that the species can respond to different water temperatures plastically, which may provide hints for its invasion success in regions with diverse climates.

## Introduction

The ability of alien species to cope with new and heterogeneous environments is essential for their successful establishment in areas outside their native ranges. For introduced species that have spread across a wide distributional range, phenotypic plasticity has often been proposed as an important contributor to invasion success^1^, and this is especially the case for invasive species with low genetic diversity^2^. *Alternanthera philoxeroides* (alligator weed), a species native to tropical regions in South America, has now invaded almost all the tropical and temperate areas around the world. Recent research has shown that phenotypic plasticity, not genetic diversity, allows *A. philoxeroides* to invade diverse habitats across broad geographic areas^2^. Furthermore, although widely distributed in China, molecular data has indicated that its genetic diversity is extremely low in China^3^, suggesting the importance of phenotypic plasticity in its invasion success. There have numerous studies showing that *Alternanthera* species exhibit high plastic responses to and are highly tolerant of flooding^4–6^, which may explain the success of *A. philoxeroides* in habitats prone to flooding. However, how *Alternanthera* copes with differences in the timing of flooding in different climate zones has not yet been studied.

Across different climate zones, the timing of flooding can be highly variable. In tropical regions, flooding usually occurs in the growing season, with high air temperatures^7^, while in temperate regions, rise of river water level prevails in winter and spring^8^. In permanent wetlands, a high water table can be maintained throughout the year^9^. Global climate change may also increase winter flooding in temperate regions^10^. Crawford^11^ stated that the strategies of winter flooding tolerance can be quite different from those of summer flooding tolerance, most likely owing to the large difference in temperature. Van Eck *et al*.^12^ found that it is water temperature, not acclimation of different plant growth stage that determines the responses of plant species to variation in flooding timing. Therefore, flooding tolerance may vary with water temperature. Effects of temperature on hypoxia/flooding tolerance have been reported in some crops^13–15^ and wild species^16,17^, but the underlying mechanisms need further investigation.

Plant species have developed diverse strategies to cope with flooding^18–20^. Flooding is detrimental to most terrestrial plants, causing reductions in growth or even death^8^. The harm caused by flooding is partly owing to carbon starvation because the leaf photosynthetic apparatuses are injured by anoxic conditions^21,22^, and the leaf assimilation rate is reduced or completely inhibited due to very weak light transmission and very low supplies of oxygen and carbon dioxide^23^. Upon submergence, some plant species rapidly accelerate shoot elongation and outgrow floodwaters and thus maintain fast gas exchange and re-establish aerial photosynthesis^24^. The benefits of shoot elongation depend on water depth, and shoot elongation is beneficial only if the flood waters are not too deep to outgrow^24,25^. In contrast, carbohydrate consumption and shoot elongation are completely inhibited in other species^25,26^. The importance of conservative utilization of carbohydrates in flooding tolerance has been shown in some tolerant rice genotypes with limited shoot elongation upon submergence^27^.

Due to the remarkable effects of temperature on plant metabolism^28^, water temperature could have large impacts at the metabolic level and on the carbohydrate consumption of submerged plants^15^ and therefore on their potential recovery from flooding stress. Water temperature may also affect the shoot elongation capacity of submerged plants. Therefore, plants may respond to different water temperatures differentially. *Alternanthera*, which has a wide distribution in different climate zones^2^, is thought to tolerate different timing of flooding with different water temperatures, but the effects of water temperature on its submergence tolerance and the possible underlying mechanisms have not been reported. In this paper, recovery growth, dynamics in carbohydrate concentration level, respiration rate, shoot elongation rate and benefits of shoot elongation for biomass accumulation are compared at different water temperatures. Since *A. philoxeroides* is able to successfully invade in different climate regions, we predict that submerged *A. philoxeroides* can respond to water temperature highly plastically, which may endow the species with high invasion potential.

## Results

At different water temperatures, the pH of the water slightly decreased with decreasing water temperature, while both dissolved oxygen and carbon dioxide increased (Table 1).

**Table 1:** Water pH, dissolved O_2_ and CO_2_ concentration at 10 °C, 20 °C and 30 °C in the containers.

Water temperature	10 °C	20 °C	30 °C
pH	7.65	8.10	8.15
Dissolved O_2_ (mg · l^−1^)	9.08	7.15	6.02
Total dissolved CO_2_ (μmol·l^−1^)	230	183	125

### Biomass accumulation during the submergence and the post-submergenc periods

**Figure 1:**
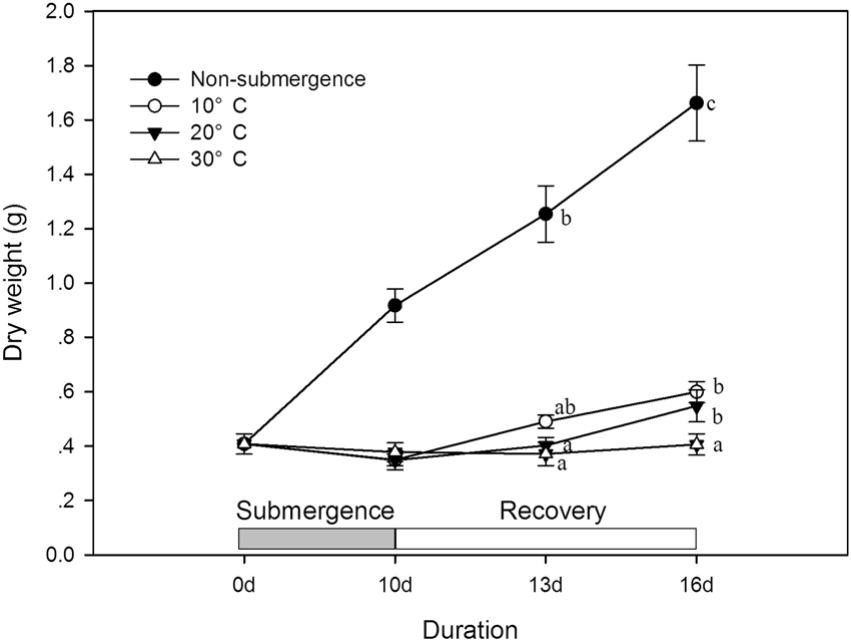
Dry mass (g) of individual non-submerged plants (closed circle) and plants submerged at water temperatures of 10 °C (open circle), 20 °C (closed triangle), and 30 °C (open triangle) at the beginning of the treatment (0d), after 10 days of submergence (10d) and after recovery for 3 days (13d) and 6 days (16d).

In the 10 days of the treatment period, the total biomass of the non-submerged plants increased remarkably, and the submergence treatments caused very little biomass loss at any water temperature (Fig. 1, P > 0.05). A difference in biomass accumulation occurred during the recovery period; previously submerged plants at lower water temperatures tended to accumulate biomass faster (Fig. 1, P < 0.05).

### Shoot elongation

**Figure 2:**
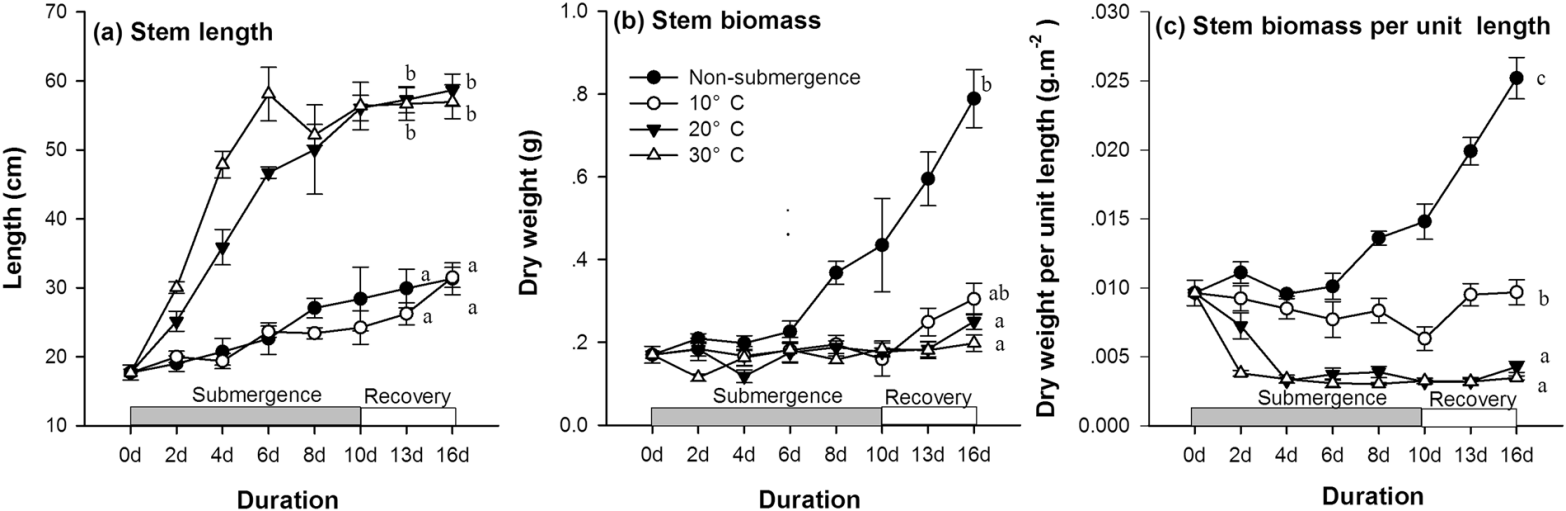
Stem length (cm), stem biomass (dry weight, g), and stem biomass per unit stem length (g cm^−1^) of individual non-submerged or submerged plants at three different water temperatures: 10 °C (open circle), 20 °C (closed triangle), and 30 °C (open triangle) at 0d, 2d, 4d, 6d, 8d and 10d.

**Figure 3:**
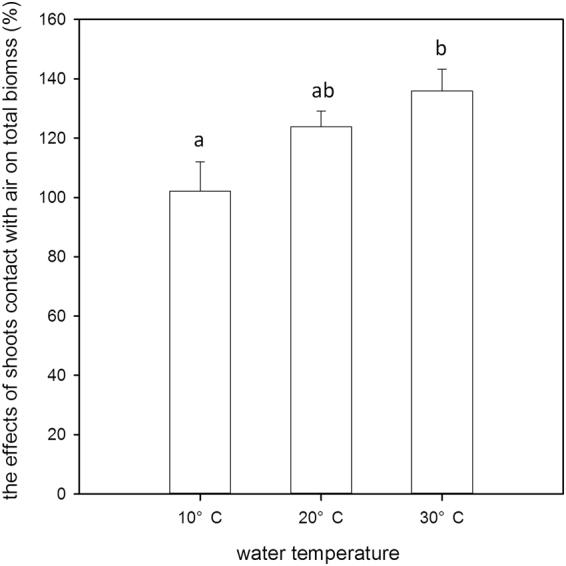
The percent (%) dry mass of individual submerged plants with contact to free air relative to plants kept under water at the corresponding water temperatures of 10 °C, 20 °C and 30 °C. All the plants were submerged for 10 days; afterwards, for half of the plants at each water temperature, the first 2 fully expanded leaves were allowed to come into contact with free air, while the other half of the plants were kept under water. Plants were harvested after 6 days of shoot exposure or without shoot exposure, and dry mass was determined.

Submergence at water temperatures of 20 °C and 30 °C induced fast upward stem elongation; non-submerged plants and submerged plants at the water temperature of 10 °C only increased slightly and even slower in the latter treatment (Fig. 2a). However, the dry weight of stems increased substantially in non-submerged plants and remained relatively stable in submerged plants (Fig. 2b, P < 0.05), and the stem dry weight per unit length consequently increased in non-submerged plants and decreased remarkably at 20 °C and 30 °C but relatively mildly at 10 °C (Fig. 2c). During the recovery period, stem length and dry mass increased in the non-submerged plants and submerged plants at 10 °C, but remained almost stable in the submerged plants at 20 °C and 30 °C. The beneficial effects of biomass accumulation were investigated by a comparison between plants kept submerged and the plants that were allowed contact with free air (Fig. 3). The relative increase in biomass when the submerged plants were allowed to have access to air increased with increasing water temperature (P < 0.05).

### Sugar content

**Figure 4:**
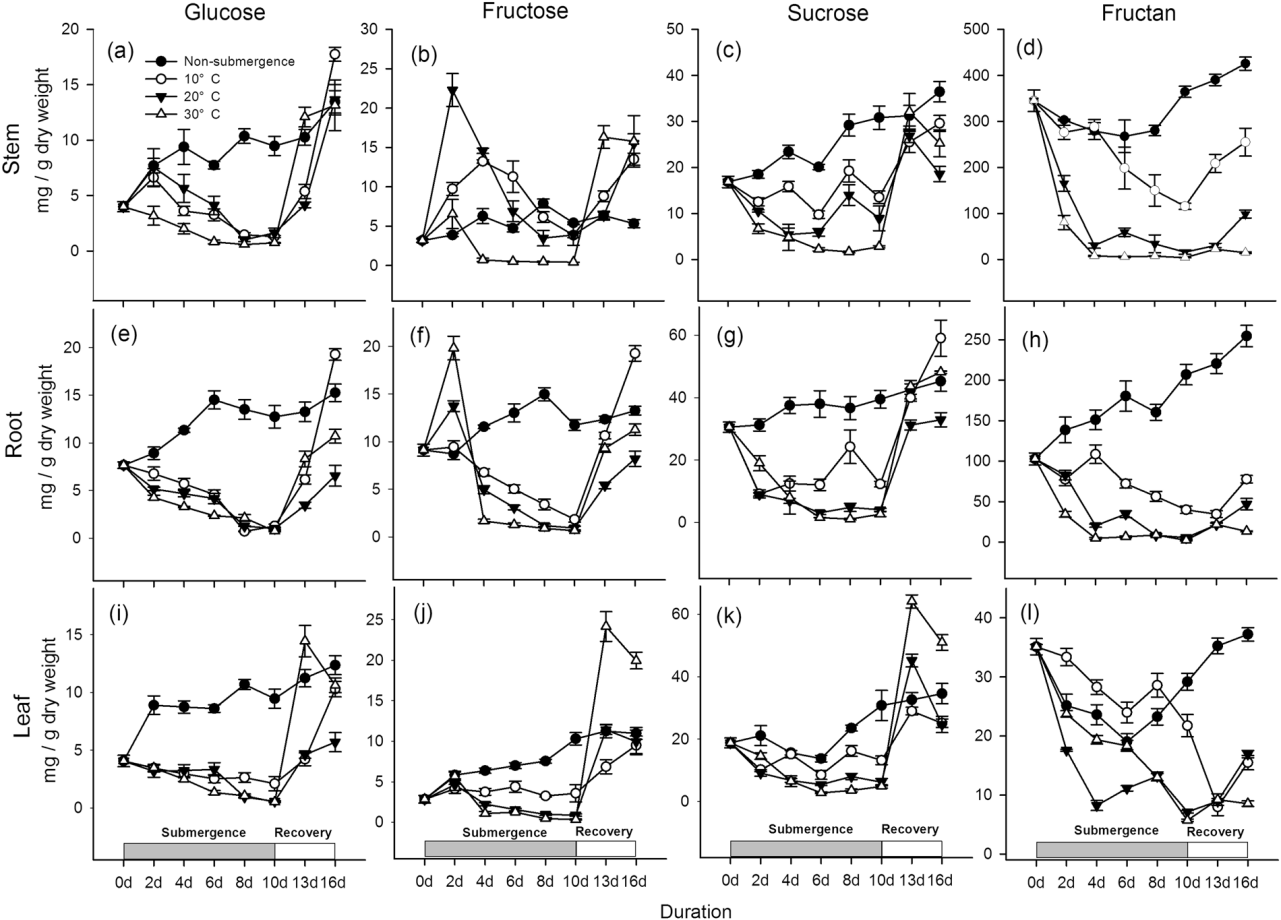
Glucose (**a**,**e** and **i**), fructose (**b**,**f** and **j**), sucrose (**c**,**g** and **k**) and fructan (**d**,**h** and **l**) concentration (mg · g^−1^ dry mass) in non-submerged plants (closed circle) and submerged plants at different water temperatures: 10 °C, open circle; 20 °C, closed triangle; 30 °C, open triangle. Different tissues were investigated separately: (**a**–**d**) for stems, (**e**–**h**) for roots, (**i**–**l**) for leaves. The carbohydrate concentrations were measured and are shown for every other day. Afterwards, starting from 10d, the plants were transferred to the normal growth conditions, which were the same as those experienced by the non-submerged plants, and the carbohydrate concentrations were measured at day 13 and day 16 (after recovery for 3 days and 6 days). Data are means ± SE, n = 5.

Submergence caused large reductions in both soluble sugar concentration and fructan concentration in *A. philoxeroides* plants independent of water temperature (Fig. 4). In submerged plants, glucose concentrations decreased in stems, roots and leaves, and the reduction in glucose concentration increased with increasing water temperature (Fig. 4a,e and i). In the recovery period, the glucose concentration increased much faster in plants submerged at a water temperature of 30 °C from day 10 to day 13. On day 16, the glucose concentration was much higher in leaves of plants submerged at 30 °C and 10 °C than at 20 °C (P < 0.05), and in stems and roots, the glucose concentration was highest in plants at 10 °C (P < 0.05).

In contrast to glucose content, in submerged plants, the fructose concentration peaked on day 2 and day 4. The peaks were very remarkable in stems and roots and less remarkable in leaves (Fig. 4b,f and j). Afterwards, the fructose concentration started to decrease until the end of the submergence period and was generally higher at lower water temperatures. The fructose concentration increased during the recovery period. From day 13 to day 16, the fructose concentration in stems and leaves increased much faster in plants at 30 °C than at 20 °C and 10 °C (P < 0.05); in roots, fructose concentration increased fastest at 10 °C and lowest at 20 °C (P < 0.05). On day 16, the fructose concentration was very similar in plants at the three different water temperatures in stems (P > 0.05); in roots, it was highest at 10 °C and lowest at 20 °C (P < 0.05); in leaves, the fructose concentration was similar in plants at 10 °C and 20 °C and higher in plants at 30 °C (Fig. 4b,f and j, P < 0.05).

The sucrose concentration was decreased by submergence as well (Fig. 4c,g and k). For plants submerged at 10 °C, the sucrose concentration decreased from day 0 to day 6, followed by a slight increase in the following submergence period (day 6 to day 10) (Fig. 4c,g and k). For the plants submerged at 20 °C, the sucrose concentration in stems decreased from day 0 to day 6 and increased from day 6 to day 10 (Fig. 4c), but in the roots and leaves (Fig. 4g and k), it decreased during the first 6 days and then remained at a low level. For plants submerged at 30 °C, the sucrose concentration in all tissues decreased from day 0 to day 6 and then remained at a low level (Fig. 4c,g and k). From day 10 to day 13, the sucrose concentration in all tissues at all water temperatures increased sharply; in stems and in roots, it was at similar levels in plants at the three water temperatures (Fig. 4c and g, P > 0.05), but in leaves, it was highest in plants submerged at 30 °C and lowest at 10 °C (Fig. 4k). On day 16, in stems and roots, the sucrose concentration was highest at 10 °C and lowest at 20 °C; in leaves, it was highest in plants at 30 °C and similar in plants at 10 °C and 20 °C (Fig. 4c,g and k, P < 0.05).

The fructan concentration decreased in all tissues at all water temperatures (Fig. 4d,h and l). It was generally higher with lower water temperatures in stems and roots (Fig. 4d and h); in leaves, the fructan concentration was highest at 10 °C but lowest at 20 °C (Fig. 4l, P < 0.05). In the recovery period, in stems and roots, it increased greatly in plants previously submerged at 10 °C and 20 °C but increased only slightly in plants at 30 °C (Fig. 4d and h, P < 0.05); in leaves, it decreased sharply from day 10 to day 13 in plants previously submerged at 10 °C but increased slightly in plants at 20 °C and 30 °C (Fig. 4l, P < 0.05).

**Figure 5:**
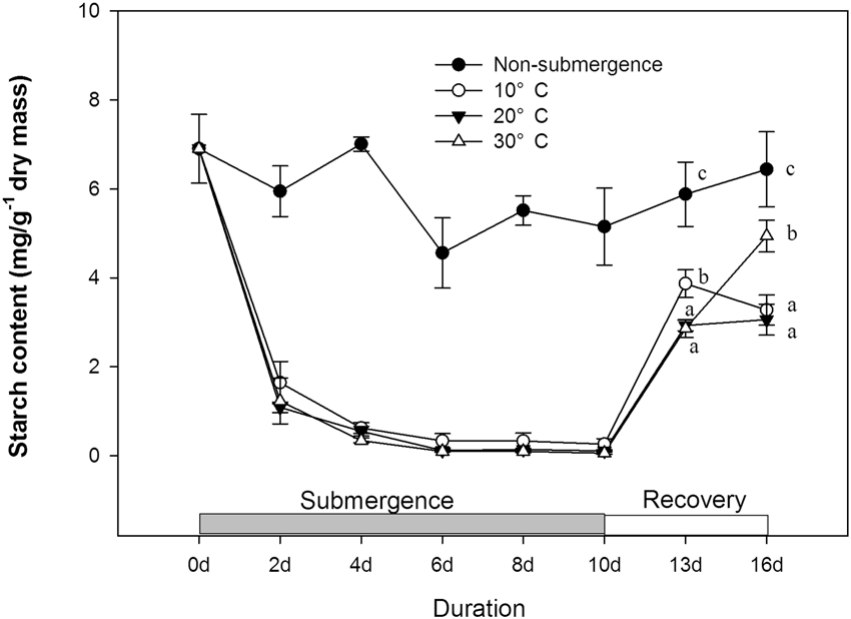
Starch concentrations (mg · g^−1^ dry mass) in leaves of non-submerged plants (non-submergence, closed circle) and submerged plants at different water temperatures: 10 °C, open circle; 20 °C, closed triangle; 30 °C, open triangle. The starch concentrations were measured and are shown for every other day. Afterwards, starting from 10d, the plants were transferred to the normal growth conditions, which were the same as those experienced by the non-submerged plants. The starch concentrations were measured at day 13 and day 16 (after recovery for 3 days and 6 days). Data are means ± SE, n = 5.

Leaf starch concentration remained relatively stable in non-submerged plants during day 0 to day 16 (P > 0.05). In submerged plants, the starch concentration decreased significantly (Fig. 5, P < 0.05) and almost at the same decreasing rate for the three water temperature (P > 0.05) during day 0 to day 10. In the recovery period, the leaf starch concentration in all submerged plants increased remarkably from day 10 to day 13 (P < 0.05) and remained stable from day 10 to day 16 for plants previously submerged at 10 °C and 20 °C but still increased for plants previously submerged at 30 °C (Fig. 5, P < 0.05).

### Respiration rate

**Figure 6:**
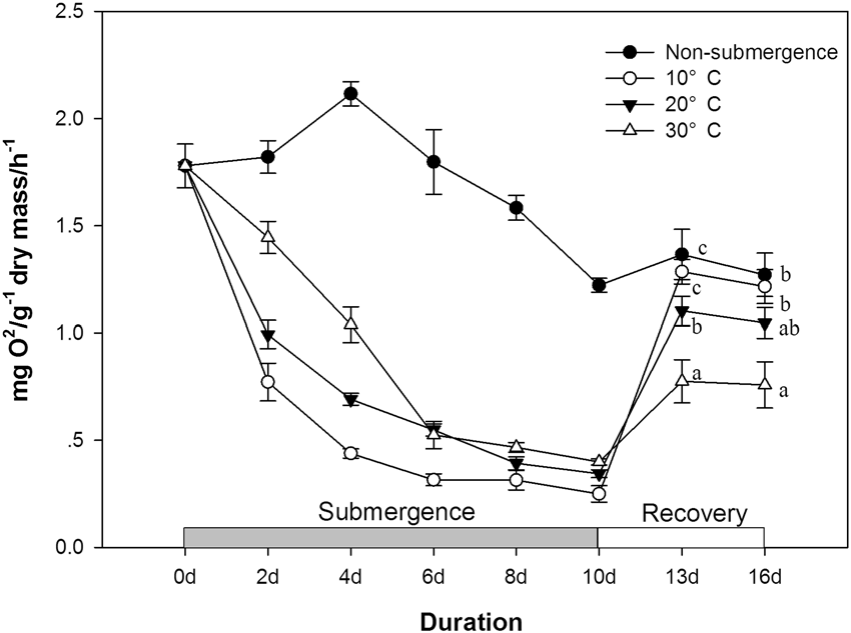
Respiration rate (mg O_2_ · g^−1^ dry mass · h^−1^) of individual non-submerged plants (closed circle) and submerged plants at three different water temperatures: 10 °C (open circle), 20 °C (closed triangle), and 30 °C (open triangle). The respiration rates were measured and are shown for every other day. Afterwards, starting from 10d, the plants were transferred to the normal growth conditions, which were the same as those experienced by the non-submerged plants, and the respiration rates were measured at day 13 and day 16 (after recovery for 3 days and 6 days). Data are means ± SE, n = 5; a total of 145 plants were used.

Submergence reduced the respiration rate at all water temperatures (Fig. 6, P < 0.06). The respiration rate level decreased with decreasing water temperature (P < 0.05). The difference in respiration rate between 10 °C and 20 °C was not significant from day 6 to day 8 (P > 0.05). During the recovery period, the respiration rate of submerged plants increased from day 10 to day 13; this increase was fastest in plants at 10 °C and lowest in plants at 30 °C (P < 0.05). From day 13 to day 16, the respiration rate in plants submerged at different water temperatures remained more or less stable but was still highest in plants at 10 °C and lowest in plants at 30 °C (P < 0.05).

## Discussion

Our results indicate that the submerged *A. philoxeroides* plants responded plastically to the different water temperatures (Figs 1–5), which may enable this species to successfully invade in different climate zones with different flooding timing patterns.

The results show contrasting effects of different water temperatures on submergence tolerance. The low water temperatures (10 °C, 20 °C) benefited the *A. philoxeroides* plants with a faster recovery growth rate, indicating higher tolerance, which was likely attributed to a lower respiration rate, slower carbohydrate utilization and a higher carbohydrate content level before returning to the non-submergence period (Fig. 6). The down-regulation of metabolism is very likely due to biochemical limitations and any growth activity at low temperatures. The conservation of carbohydrates and energy may confer on the species a high tolerance of long-term submergence or anoxic conditions^11,29^. In contrast, the biomass accumulation of de-submerged *A. philoxeroides* plants at 30 °C was much slower. The negative effects of high water temperature on flooding tolerance was likely due to the fast carbohydrate consumption induced by the high respiration rate and fast shoot elongation (Fig. 4). Although *A. philoxeroides* was less tolerant of submergence at 30 °C, its shoots elongated quickly, which benefited the submerged plants more at 30 °C when the shoots again came into contact with air (Fig. 3), suggesting that shoot elongation is an effective strategy for coping with submergence if the water depth is shallow and the temperature is high. However, it has also been demonstrated that shoot elongation is costly in energy and carbohydrate reserves^27,30^. Therefore, if the water is too deep, excessive shoot elongation may spend too much energy and lead to plant death^31^.

The results suggested that many processes involved in submergence tolerance are regulated by water temperature. First, carbohydrate metabolism, which involves gene expression and enzyme activities, is strictly controlled by temperature^28^. Therefore, fructan and starch degradation and respiration are both determined by temperature. Second, shoot elongation under water also depends on temperature (Fig. 2). It has been reported that shoot elongation stimulated by submergence is regulated by several plant hormones^32^ and depends on carbohydrate reserve mobilization^33^. Therefore, the effects of water temperature on shoot elongation may be attributed to its effects on the processes of either hormone regulation or carbohydrate utilization. In the present study, carbohydrate utilization slowed down at a water temperature of 10 °C, which may explain the inhibited shoot elongation at this low temperature. The fast shoot elongation of *A. philoxeroides* at 30 °C may indicate adaptation to its original tropical habitats, where high temperatures favour this escape strategy. Whether the plastic responses to water temperature are unique in this *A. philoxeroides* or common in many species needs further study.

The major non-structural carbohydrate reserve in *A. philoxeroides* stems is fructan (Fig. 4). Fructan, as an energy store, was mobilized quickly in response to submergence (Fig. 4), providing energy for both fast shoot elongation and maintenance under the circumstances of restricted photosynthesis. Compared with starch, some studies have noted that fructan may play important roles in adaption to oxygen deficiency due to the advantages of the lower energy cost of the storage of sucrose in the form of fructose polymers and little negative feedback in the photosynthetic apparatus^34^. All these results suggest the importance of fructan storage mobilization for short-term submergence tolerance in *A. philoxeroides* plants.

Our results have implications for the invasion success of *A. philoxeroides* plants in diverse areas around the world. *A. philoxeroides* is highly plastic in its responses to water temperatures; although the high water temperatures in tropical areas decrease its potential recovery from submergence and endow the plants with the advantage of restoring contact with air under the circumstances of shallow water, the low water temperatures in temperate regions in the non-growing season may benefit the species with little carbohydrate and energy utilization and improve its tolerance of long-term deep-water submergence. The enhanced growth during recovery suggests that *A. philoxeroides* can potentially invade regions with a cooler climate. In conclusion, the plastic responses to different water temperatures may partly explain the invasion success of *A. philoxeroides* in regions with variable climate regimes.

## Plant Materials and Methods

### Plant material and growth conditions

*Alternanthera philoxeroides* (Mart.) Griseb, commonly called alligator weed, is native to South America but has spread to most parts of the world. In China, it is one of the most noxious invasive species due to its fast growth rate. A previous study^4^ (Wang *et al*. 2008) found that the *A. philoxeroides* plants from the Three Gorges Reservoir region could tolerate up to six months of complete submergence. Therefore, it can act as a pioneer species, colonizing the water fluctuation zone when the water recedes at the end of spring. A single clone of *A. philoxeroides* was collected from the riparian area of the Jialing River, a branch of the Yangtze River in September 2007. The clone was transported to and cultured in a greenhouse. New plants were propagated from this single clone and transplanted to pots (380 cm^3^) filled with one part potting soil and one part sand. The plants were grown for a further 14 days in an indoor laboratory where the growth conditions were well controlled, with a constant air temperature of 20 °C, photosynthetically active radiation (PAR) of 102 µmol photons · m^−2^ · s^−1^ and a light period of 16 h. The light intensity was measured with a light sensor (X1_2_ optometer, Gigahertz-Optik, Germany).

### Submergence treatments at different water temperatures

Just before the treatments began (day 0), some of the plants were harvested to determine the initial respiration rate, stem length, dry mass and carbohydrate concentration (day 0, n = 5). One group of plants was regularly watered, and the soil was well drained, providing supplemental information on non-submerged plants. The other three groups of plants were completely submerged in six plastic containers (20 plants each, 250 l, 80 cm water depth) filled with tap water. The water in the containers was saturated with air by the continuous and gentle pumping of air into the water. The water temperature was regulated at 10 °C, 20 °C or 30 °C, with two replicate containers per water temperature. For the 30 °C treatments, the water temperature was maintained using two heating sticks, while the 10 °C water temperature was maintained by circulating cooled water in plastic tubes in the containers. The illumination just above the water surface was the same as for the non-submerged plants. Every other day (days 2, 4, 6, 8 & 10), whole individual plants from each water temperature treatment were harvested for stem length, respiration rate, dry mass and carbohydrate concentration analysis (n = 5).

### Recovery from submergence

After ten days of the submergence treatments, the remaining plants from each submergence treatment at the three water temperatures were returned to non-submergence conditions that were the same as for the non-submerged plants. Harvesting from the three previously submerged groups of plants as well as non-submerged plants occurred after three and six days of recovery (day 13 and day 16); respiration, dry mass and carbohydrate concentration were measured as before.

### Stem length measurement

For each individual plant, the length of the main stem was calculated as the sum of all internode lengths. Later, the dry weight of the individual stems was measured, and the stem dry weight per unit stem length was calculated.

### The beneficial effect of shoot contact with air on biomass accumulation

Shoot contact with free air could benefit submerged plants by resulting in less biomass loss or additional biomass accumulation. The total biomass of individual plants was compared between completely submerged plants and submerged plants with shoot contact with air. Five plants were continuously submerged for 16 days, while another five plants were submerged for 10 days, and in the following 6 days, two pairs of fully expanded leaves were allowed to have contact with air by lifting the pots upward. The PAR was 102 µmol photons · m^−2^ · s^−1^, and the light period was 16 h.

### Respiration rate estimation

The plants (or the tissues from the additional plants harvested on day 10) were rinsed under tap water, and the respiration rate was immediately measured. Respiration activity was estimated as the oxygen uptake rate of a single entire plant. The plants (tissues) were enclosed in airtight glass bottles fully filled with 1.1 l tap water. The water was air saturated before measurement (O_2_: 7.2 mg · l^−1^, total inorganic carbon: 170 mg · l^−1^), with a constant temperature of 20 ± 0.1 °C. An oxygen sensor (the same type as above) was inserted into the bottle through a rubber stopper. The system was checked for leakage prior to the measurements. The incubation water was stirred vigorously using a magnetic stirrer to homogenize the oxygen concentration. The oxygen concentration in the bottle was monitored by the sensor, which was connected to a data logger. The incubation duration was 20–30 min. This incubation was short enough that the respiration rate was not limited by the decreasing O_2_ concentration. The pH of the incubation water decreased during incubation, but this reduction was always less than 0.3 points. The respiration rate of a whole plant or any tissue was calculated as

$${\rm{Oxygen}}\,{\rm{uptake}}\,{\rm{rate}}=\frac{({C}_{\mathrm{O2}(0)}-\,{C}_{{\rm{O2}}({\rm{t}})})\times V}{t\times DM}$$

*C*_O2(0)_: initial O_2_ concentration (mg/L) in the water (just before incubation)

*t*: incubation duration (hour)

*C*O_2(*t*)_: final O_2_ concentration (mg/L) in the water (just after incubation for a period of t)

*V*: water volume (L)

*DM*: dry mass (g) of the whole plant or tissue

### Carbohydrate analysis

The harvested plants were washed, and after 1–2 hours of material processing, the plant samples were immediately frozen and freeze dried, and the different parts of the plants (leaves, stems and roots) were weighed and ground into powder. The glucose, fructose and sucrose concentrations were measured using an enzymatic method described by Jones *et al*.^35^. The presence of starch was checked with amyloglucosidase and α-amylase (Roche Diagnostics GmbH, Germany). The results showed that starch was found only in the leaves of the *A. philoxeroides* plants, while the starch concentrations in the stems and roots were under the detection limit of the method (<1 mg · g^−1^ dry mass), indicating the absence of starch. These two tissues could therefore be disregarded. Next, another important polysaccharide, fructan, was evaluated using an enzymatic fructan assay kit (Megazyme Ltd., Ireland); as we found a higher amount of fructans than starch in the plants, the fructan concentrations in various plant parts were therefore analysed using the same fructan assay kits.

### Data analysis

All the statistical analyses were conducted using Sigmastat 2.03 (SPSS Inc, Chicago). The effects of duration and treatment (non-submergence, submergence at different water temperatures) on carbohydrate (glucose, fructose, sucrose, total fructans and starch) content, respiration rate and stem length were statistically analysed by two-way ANOVA or the Kruskal-Wallis test. When necessary, the effects of duration within each treatment or the effects of the treatments within each sampling date were evaluated with one-way ANOVA. Multiple comparisons between the treatments were conducted to reveal the differences among the different water temperature treatments. The beneficial effects of shoot contact with air were evaluated with one-way ANOVA. The significance level was set at P = 0.05.

### Data availability

All data generated or analysed during this study are included in this published article (and its Supplementary Information files).

## Electronic supplementary material

Dataset 1
